# Influence of Mental Emotion in the Production of Disease of the Heart

**Published:** 1851-07-01

**Authors:** W. Fraser


### INFLUENCE OF MENTAL EMOTION IN THE PRODUCTION OF DISEASE OF THE HEART.

BY W. FRASER, ESQ., M.R.C.S.E.

(Read at the Medico-Chirurgical Society, Aberdeen.)

The subject of the following remarks was an unmarried female, forty-four years of age, whom I attended for the first time about a year ago. She was then labouring under decided symptoms of valvular disease of the heart, which had been of considerable (I suppose about five or six years) standing. The change which she had shortly before then undergone, from a private to a public position, involving considerable responsibility, and requiring an amount of activity which she had never been accustomed to, and which she was then unable to render, together with the consideration that the original causes which had probably led to the disease still continued in operation,—all tended to the formation of a guarded and, indeed, an unfavourable prognosis; and it was in accordance with this view that my opinion was given of the case.

By the use of a diuretic and tonic plan of treatment, and the retirement to the country, so as to ensure the most perfect withdrawal of mental and physical excitement, she at that time perfectly recovered; that is to say, the more obvious and distressing symptoms, such as the anasarca, hydrothorax, dyspnœa, cough, extreme debility and exhaustion on the slightest muscular exertion, had disappeared, although, of course, the disease itself could not be supposed to have been removed. In fact, notwithstanding every precaution that her situation admitted of, it began again in a few months to develop itself in the usual results attending valvular obstruction of the heart, although not to the same extent that had occurred on the previous occasion. About six weeks before her death she had a sudden and violent attack of common cholera, from the prostration occasioned by which she never properly recovered. This might have been considered as an effort of nature to relieve the overloaded tissues and vessels; which effect it had to a considerable extent, and she so far rallied that great hopes were entertained that she would soon be restored to her usual condition, till one day, through her own and her attendant's inadvertence, she left her bed-room, and was placed at the fireside of a large room, an open window of which transmitted a draught of air directly athwart her person. The consequence of this indiscretion was an attack of pneumonia, which, although all along masked and overlaid, as it were, by the gravity of the previously established symptoms, evidently was the means of bringing the case to a fatal termination. Dr. Ogston attended her with me for the last fortnight, and he and Dr. Steel were present with myself at the autopsy on the 21st of the month of February.

Not having taken any notes either of the history or post-mortem examination of the case, I must confine myself to such remarks as my memory will sufficiently warrant me in making. In the chest, about the lower third of the right lung was hepatized (and partially adherent to the costal pleura), and there were old adhesions between its middle lobe and the wall of the chest; the lungs throughout were rather more than usually gorged, and there was a quantity, not large, of fluid in both cavities of the thorax. The heart, which was abundantly covered with fat, appeared to be of the normal size: it contained but little blood, and its muscular substance seemed tolerably firm and healthy. All its cavities, but more especially the left ventricle, contained pieces of pure, pale, and firm fibrin, which was in some places so intermeshed with the chordæ tendineæ and calumnæ carneæ, as to be with difficulty removed. The auricles, but particularly the left one, were pale and attenuated; the ventricles, on the other hand, especially the left one, were very considerably contracted in their capacity and hypertrophied in substance. The semi-lunar valves, at the origin of the pulmonary artery, were thickened, and must have been impaired in their function, as they did not prevent the passage of water when it was poured into the artery, though this was probably in part owing to recent fibrinous deposit. The mitral valve, however, presented the greatest marks of disease, as will be shown by the preparation. Its orifice was of the button-hole shape, and measured about three-fourths by one-fourth of an inch in diameter. The contraction produced by osseo-cartilaginous deposit was such that it could scarcely admit the tip of the little finger, instead of the two forefingers, as the opening, when of its natural size, will do. Its flaps were so thickened and altered in shape by the deposition within their substance of a sort of brittle osseous or osseo-calcareous matter, that they must have permitted a considerable regurgitation of blood into the auricle.

The abdomen, from which more than half a bucketful of bloody serum was removed, was not very minutely examined. The liver was found slightly enlarged and indurated. The spleen was likewise indurated, and the kidneys were pale and very deteriorated in the cortical substance.

Having sent this paper to Dr. Ogston, with a request that he would correct any inaccuracies in my account of the post-mortem appearances, he sent me the following reply, which, I think, will show that there is no material discrepancy between our recollections of the case; and moreover, from the well-known accuracy and extensive experience of the Doctor in this department, will be a guarantee as to the general correctness of the description.

"Adelphi, 4th March, 1851.

"Dear Sir,—These are my recollections of the morbid appearances in Miss ——'s case, so far as our hurried inspection allowed us to examine the body. If it serves your purpose, it is at your service.

"I am, dear sir, yours faithfully,

"(Signed) F. Ogston.

"1. Emphysema of the upper lobes of both lungs.
"2. Old adhesions of the middle lobe of the right, and the lower lobe of the left lung to the walls of the chest.
"3. Copious deposit of fat under the investing membrane of the heart.
"4. Dilatation and attenuation of the right cavities of the heart.
"5. Concentric(?) hypertrophy of the left ventricle.
"6. Contraction and old thickening of the mitral valve.
"7. Cartilaginous thickening of the tricuspid valve.
"8. Patency of the semi-lunar valves of the aortic and pulmonary arteries.
"9. Slight hypertrophy of the liver.

"10. Hepatization of the spleen.
"11. Wasting and pallor of the secreting portion of the kidneys (with albuminous urine).
"12. Moderate effusion of serum into the chest.
"13. Copious effusion of serum into the abdomen.
"14. Unusual smallness of the stomach."

As the case presented nothing unusual either in its history, progress, or treatment, and as I had preserved no notes on the subject, I shall, without entering further into these points, come at once to my object in bringing it before you.

The occurrence of so well-marked a case of valvular disease of the heart, mainly brought on, as I believe it to have been, by mental causes, having led me into a train of speculation as to the pathogeny—the modus operandi or rationale of the production—of this class of cardiac diseases, I shall, with your leave, read over a few ideas I have noted down, crude and hasty though I admit them to be.

An examination of the mechanism of the great central organ of the circulation will give one (at all events it has given me) the impression that the action and office of the mitral valve, the left auriculo-ventricular opening, is of a different nature from those of the other valves, both in the cardiac and the venous systems. While these are mere passive resisters of the return or retrograde passage of the blood, it would appear that, in addition to this function, the mitral valve, the ostium arteriosum, acts as a governor or regulator of the amount of blood which is transmitted through its opening. This it effects by the action of the calumneæ carneæ, chiefly those belonging to the larger flap of the valve. It is evident, from the construction and arrangement of the parts, that the degree to which these columns contract and relax, will decide the size of the auriculo-ventricular aperture, and of course regulate the volume of blood transmissible through it and through the vascular system generally. The exigencies and demands of the system, or constitution itself, in all the various circumstances in which it may be placed, will be the procuring or deciding cause of this action, and upon it will depend, in a great measure, the volume or fulness of the systolic or arterial pulse. The nervous influence communicated by the appropriate portion of the ganglionic system, with its various anastomoses, connecting it with the cerebrum and the most important organs of the body, may be presumed to be the means by which the muscles of the valve are, through the medium of their motor, or muscular nerves, signalled as to the extent of contraction or relaxation they shall undergo. The feelings and emotions of the mind, by whatever means they may be called into action, concentrated or collected, in all probability in some portion of the medulla oblongata, and transmitted through a branch of the par vagum, are thus brought in an instant to bear upon the actions of this important valve. Of course, it is understood, that, with its action, all other associated and independent operations, such as those of the other portions of the heart, the diaphragm, and the arterial system generally, are, through the medium of the great sympathetic nerve, brought into harmony and co-operation. But this function of the valve appears to be the first—the primum mobile—the starting-point, as it were, of this circle of vital actions, and, like the *point d'appui* of a bowstring, it is subjected to an amount of excitement, resistance, and wear, which does not bear upon any other portion of the circulatory system. The natural consequence of this will be, in accordance with what we see in other parts of the body when subjected to a long course of irritation and over-action, that the organ will become strengthened and thickened by the deposition of additional substance, either of a homogeneous or of an analogous kind, and this deposition, under the continued operation of the cause that produced it, will go on till the proper configuration and action of the part become very much impaired, or even at last entirely destroyed.

Persons who by practice have acquired the power of paying minute attention to their nervous and internal sensations (a very unadvisable and dangerous habit, by the way, especially in this instance), can easily recognise and feel the action of which I have been speaking; and they can even at times, by an effort of the imagination and the will, voluntarily cause it to take place. The mode in which this can be done, speaking from my own slight experience, is this:—supposing the patient, or experimenter rather, to have been for some time gently occupied in body and mind, as in a reverie or waking dream, for instance, so as to be generating a larger amount of nervous influence than he is expending, let him quickly call before his mind's eye some scene or circumstance, or combination of events, calculated to produce a feeling of consternation—a sudden emotion of fear, grief, and surprise—and he will—or rather he may, under certain conditions that can scarcely be described—feel a kind of aura or tremor proceed from the base of his brain down to the heart. Immediately a sort of spasmodic closure of the valve is felt to take place, giving a sensation like the sudden contraction of an involuntary sphincter muscle, and causing, by the impulse of the arrested blood, a violent jerk or leap of the heart at the same time, as a natural result of the arrest of the current of blood thus occasioned, a short flutter or struggle of the overloaded left auricle takes place, till the entire organ has had time, by the increased rapidity and force of its action, induced probably in part by the stimulus of over-distention, to make up for, and accommodate itself to, the diminution in the current of its contents.

But here the question naturally presents itself—as nature, or the vis medicatrix naturæ, does nothing in vain, or without some beneficial purpose in view, what is the object of the action under discussion, resulting, as we know it in some circumstances to do, in the most morbific and disastrous results? In the beautiful language of Shakspeare we might ask—

> "Why does my blood thus muster to my heart,
> Making both it unable for itself,
> And dispossessing all the other parts
> Of necessary fitness?
> So play the foolish throngs with one that swoons;
> All come to help him, and so stop the air
> By which he should revive; and even so
> The people subject to a well-wished king
> Quit their own part, and in obsequious fondness
> Crowd to his presence, where their untaught love
> Must needs appear offence."
>
> *Measure for Measure*, Act ii. scene 4.

And again:

> "Even such a passion doth embrace my bosom:
> My heart beats thicker than a feverish pulse;
> And all my powers do their bestowing lose,
> Like vassalage at unawares encountering
> The eye of majesty."
>
> *Troilus and Cressida*, Act iii. scene 2.

This action, I will venture to reply, like every other natural process in the animal economy, is essentially and in itself of a healthful and preservative kind, and it is only by being thwarted and diverted from its original purpose, that it issues in that series of baneful results of which it is often the first link. I have observed, on microscopically examining an insect in which the action of the heart and blood-vessels could be seen, that on receiving a wound, the first and almost instantaneous effect produced is a spasmodic contraction of the heart, followed by an unusually long and large dilatation; and, afterwards, a more rapid, and at first almost convulsive action of the organ: the blood is, at the same time, seen to be directed with increased force and abundance towards the wounded part, and the animal puts itself into an attitude of defence, or perhaps of retaliation and attack.

Precisely the same thing takes place, in similar circumstances, in the human body—the instinctive or vital provisions for self-preservation and defence being the same in both cases. To borrow the idea of Shakspeare, in a passage which I shall presently quote, in reply to the question that we asked in the same poet's words—it may be said, that on the instant of danger occurring or being discovered, a message is telegraphed to the citadel, from which reinforcement and succour are to be looked for: there the alarm is immediately sounded, the portcullis is closed, or at least put under guard, the forces are mustered, and, amidst the turmoil of preparation and arrangement, a momentary consultation is held, and then the required assistance is despatched to the region in want of it.

> "Between the acting of a dreadful thing
> And the first motion, all the interim is
> Like a phantasma, or a hideous dream:
> The genius and the mortal instruments
> Are then in council; and the state of man,
> Like to a little kingdom, suffers then
> The nature of an insurrection."
>
> *Julius Cæsar*, Act ii. scene 1.

But besides the attacks of mere brute force, and the palpable evidence of immediate jeopardy to his bodily safety, man,—a creature of intellect and feeling,—can easily recognise and appreciate the existence of other dangers that equally menace his happiness and life: and here, too, the same instinctive process of warning and of self-defence that we have been considering, is still employed by the vis medicatrix naturæ, although its external manifestation will differ in accordance with the nature of the threatened danger. "Sorrow," says Melancthon, "strikes the heart as with a blow, and causes it to languish with a deep feeling of pain." Such would probably have been the case in one of his intellectual and angelic temperament, while in those of a different constitution, like his friend, the energetic Luther, a different result would have been produced. The outpouring of speech, for instance, is one of the most obvious means had recourse to; as Shakspeare again says—

> "The heart hath treble wrong
> When it is barred the aidence of the tongue.
> An oven that is stopped, or river staid,
> Burneth more hotly, swelleth with more rage:
> So of concealed sorrow may be said."
>
> *Poems.*

And on another occasion:—

> "Give sorrow words; the grief that does not speak
> Whispers the o'erwrought heart, and bids it break."
>
> *Macbeth*, Act iv. scene 3.

Or, the whole system may be roused into a fit of passionate excitement, and this with a very beneficial effect as regards the individual's personal health and comfort; for as Armstrong, the poet of medicine, says—

> " There are meantime to whom the boisterous fit
> Is health, and only fills the sails of life:
> For where the mind a torpid course would run,
> And each clogged fountain lazily move on,
> A generous sally spurns th' incumbent load,
> Unlocks the breast, and gives a cordial glow."

Or, as Akenside, still more apropos to our subject, remarks—

> " Passion's fierce illapse
> Rouses the mind's whole fabric with supplies
> Of daily impulse; keeps the elastic power
> Intensely poised, and polishes anew
> By that collision, all the fine machine.
> Else rust would rise, and foulness by degrees
> Incumbering choke at last what Heaven designed
> For ceaseless action and a round of toil."

But amidst the high-pressure conventionalities and law-protected arrangements of civilized society, it is in general the intellect or brain to which the alarm-struck heart applies—at which it may be literally said to knock—for assistance and advice. If the intellect and resources of the individual be able to extricate him from the difficulty in which he is placed, all will be well, and the instinctive actions of his system have answered the purpose of their implantation. But where they fail to do this, and when the cause of the disturbance continues constantly renewing its operation on the body, it is then that the valvular affection with all its concatenation of abnormal products may be the result. Such a fixed and irremovable heart-sorrow, for instance, was that of the majestic Lear, who thus bewails the mischief done by his ungrateful daughter, in whose power he had placed his noble and too confiding heart.

> " She hath
> Looked black upon me; struck me with her tongue
> Most serpent-like, upon the very heart."

> " She hath tied
> Sharp-toothed unkindness like a vulture here."
> *King Lear*, Act ii. scene 4.

And how significant are the words, whether taken figuratively or literally, that were written by that wise king, whose last act, also, according to the *authentic* record we have of *his* life (1 Kings, xi. 4), proved so melancholy a commentary on his own aphorism: " Keep thy heart with all diligence, for out of it are the issues of life."—Prov. iv. 23.

The dangerous and often fatal effects of the sudden removal of the cause of a prolonged heart-struggle, if the precaution of an appropriate diverticulum be not employed, are well known. May not the immediate cause be, that the left ventricle is overpowered and paralyzed, and its action thus brought to a stand, by the rapid and continuous rush of blood permitted by the too sudden relaxation of its valve? Pericles, Prince of Tyre, who had suddenly recognised and recovered his long lost daughter, thus speaks to his friend:—

> " O! Helicanus, strike me, honoured sir;
> Give me a gash, put me to present pain;
> Lest the great sea of joy rushing upon me,
> O'erbear the shores of my mortality,
> And drown me with their sweetness."
> *Pericles*, Act v. scene 1.

Hence the propriety of gradually breaking the force of good news by the interposition of a certain amount of doubt or uncertainty, and thus preparing the system for its operation; in the case of a person in whom a long course of grief has had time to reduce to a settled condition those changes which this is capable of producing on the system. The relief which the instinctive tearing or beating of the breast—the natural language of violent grief—would seem to afford, is to be accounted for on a similar principle, namely, that of establishing a diverticulum or by-path to the nervous influence, which would otherwise be transmitted to the seat of disease. On the same principle we may account for the great relief which is afforded in chronic or fixed disease of the heart by the insertion of a seton—a chronic or permanent gash—over the region of the heart.

The consecutive lesions of structure, after the valvular affection has been established, that would naturally take place in the other parts of the cardiac apparatus, and the system generally, would depend, to a great extent, on the constitutional character and temperament of the individual. The most natural and obvious consequence, in the first instance, would be, that the left ventricle would become hypertrophied without dilatation; that is to say, that its walls would become thicker and less capacious by adapting themselves to the increase of work which the more rapid, though smaller supply of blood, would entail upon them. The more passive character of the office of the left auricle, that of a safety or supply-chamber to the force-pump of the ventricle, and its engorgement with blood in the circumstances supposed, would, in general, rather lead to a dilatation of *its* cavity, with a thinning, or, at all events, without any hypertrophy of its walls. But supposing the person to be of an ardent and determined temperament, with passions yet uncooled by age, or the discipline of experience or of religion, or to be under the necessity, though of an opposite temperament, of exerting his muscular and arterial system in strong corporeal labour; when under the influence of the causes which we have supposed as tending to contract the ostium arteriosum, the probability would be, that long before the last degree of thickening or ossification of the valve could be produced, a fatal dilatation or aneurism of some other part of the cardiac or arterial system would ensue,—that of the left auricle, for instance, or of the origin, or of some neighbouring part, of the aorta.

The effect produced by the continued or frequently-repeated contraction of the mitral orifice on the venous circulation, tracing it backwards from the point of obstruction, would be, in the first place, as we have seen, an engorgement and dilatation of the ventricle; then beyond that, there would be a corresponding engorgement and retardation of the blood in the vessels of the lungs, occasioning, as it does, one of the most serious and distressing symptoms in these complaints; namely, serous infiltration and dyspnœa; beyond that, again, the right ventricle and auricle would feel the effect of the impediment, either with or without undergoing hypertrophy, dilatation, or other alteration, according to circumstances; beyond that, again, the whole venous circulation would be kept back, and its vessels, both large and small, brought into a state of congestion. Tracing the remora still further back, we come to the capillary system of arteries, and from them to the larger trunks, and finally to the aorta itself, which brings us almost to the point from which we started. The contractile force of the left ventricle, together with such action as the arteries themselves are capable of exerting, especially when under stimulation either of an emotional, therapeutic, or exertional kind, meeting, in the circumstances we have supposed, with the resistance which the obstructed capillaries interpose, would expend itself upon their own walls, and whatever portion of these was the most powerfully assailed, or happened, from whatever cause, to be unusually weakened, would naturally give way and suffer dilatation. That this would probably be at the origin of the aorta, or in some of the larger trunks, which are acted on by a large mass of blood in close proximity to the vis a tergo, rather than in the more remote and smaller branches, could easily be shown upon the established principles of hydraulics.

